# Prevention of occupational dysphonia in scientific university personnel: A cross-sectional study

**DOI:** 10.1007/s10389-022-01805-0

**Published:** 2023-01-13

**Authors:** Christiane Lücking

**Affiliations:** Department Onlineplus, University of Applied Sciences Fresenius, Alte Rabenstrasse 1, 20148 Hamburg, Germany

**Keywords:** Prevention, Health promotion, Occupational dysphonia, Voice disorder, University staff, Professor

## Abstract

**Aim:**

The aim of the study was to identify occupational and individual factors that influence the occurrence of voice problems in university staff and to investigate whether there is a link between voice problems and physical, psychological or functional changes?

**Subject and methods:**

The voices of university teachers are exposed to increased stress. As members of the group of professional speakers, they have an increased prevalence of developing a voice disorder, so-called dysphonia. In the worst case, chronic, occupational dysphonia can occur. In an empirical-quantitative study in form of a cross-sectional study in Germany, an online questionnaire was used to determine whether university teaching staff is more frequently affected by voice problems than their colleagues in the administrative sector.

**Results:**

The results show that dry room climate, background noise, poor spatial acoustics, lack of breaks, and increasing age influence the occurrence of voice problems in university teachers. Teaching staff is therefore more frequently affected by impaired vocal function due to frequent throat clearing/coughing and a raspy, hoarse and scratchy voice than their administrative colleagues. In addition, there is a need for regular training and counselling.

**Conclusion:**

In order to prevent the development of occupational dysphonia among university teachers, diagnostic, practical and theoretical interventions for the target group are needed. To this end, the appropriate structural and personnel conditions must be created in the university environment.

**Supplementary Information:**

The online version contains supplementary material available at 10.1007/s10389-022-01805-0.

## Introduction

A healthy, resilient voice is not impaired in its function, when free of background noise and faulty states of tension. This is particularly important for professional speakers, such as lecturers, who rely on a resilient voice on a daily basis. Various studies confirm evidence of an increased prevalence of voice disorders and occupational risk factors in the clientele of professional speakers, which favours the development of dysphonia (Vilkman 2004; Kooijman et al. 2006). The higher the occupational voice strain, the greater the risk of the development of occupational dysphonia caused by it. Heidelbach (1994) found evidence that the frequency of vocal problems among professional speakers increases with the length of their employment. Preparation for the expected vocal strain is often not done (Allhoff and Allhoff 2021). To make matters worse, knowledge about voice disorders, their consequences and risk factors is only rudimentarily developed, if at all (Hammer and Teufel-Dietrich 2017).

### Prevalence in the clientele of university teachers

University teachers are professional speakers and belong to the risk group of developing a voice disorder as a result of their job (Tormin and Bock 2018). They are a particularly vulnerable group due to their above average high level of speaking in their everyday work and the demands placed on their voice quality. At the end of 2018, around 400,100 people were employed as scientific staff at German universities and university clinics. This included around 47,900 professors who taught and researched at German universities and universities of applied sciences (Statistisches Bundesamt 2018). Many studies found evidence of an increased prevalence of voice disorders due to excessive speech strain. While the frequency of voice disorders in the general population is around 6%, professional speakers suffer from it at two to three times the frequency (Fritzell 1996; Sliwinska-Kowalska et al. 2006; De Jong et al. 2006). In the study by Higgins (2006), 45% of the university teachers surveyed stated that they had a voice disorder. Of these dysphonic speakers, 6.7% were chronic, that is, showing a dysphonia that lasts for more than 4 weeks, and 93.3% were acute (Higgins 2006). A systematic review and meta-analysis by Azari et al. (2022) reported an overall prevalence of voice disorders in university professors of 41%.

### Risk factors

Despite the overall high prevalence, only a few risk factors have been identified that have a significant influence (Korn et al. 2018). One influencing variable is gender: here, a gender difference to the disadvantage of female lecturers shows that females are significantly more frequently affected by voice complaints than their male colleagues (Korn et al. 2018; Gomes et al. 2018; Kyriakou et al. 2018). Perceptions of voice problems and stress are also cited as influencing factors (Korn et al. 2018). Female university faculty members are more likely to perceive their vocal problems when teaching and perceive their working conditions as more stressful (Korn et al. 2018). According to observations of Korn et al. (2015), female university teachers who reported being stressed or nervous were also more likely to experience vocal problems. Upper respiratory tract infections were found to be significantly more common among university teachers (Higgins 2006; Korn et al. 2015), and a relationship between hoarseness in teaching staff and the work environment in terms of loudness, noise levels and poor air quality has been found (Korn et al. 2015). The louder the area we are in, the louder we try to make our voices so that we can still be heard. This noise can be caused by human voices, such as by seminar participants, but also from the interaction of functional noises of projectors, air conditioning systems and other devices (Nollmeyer 2012). Lecturers who have not learned any techniques to generate the necessary vocal force often speak with too much force and develop hyperfunctional dysphonia and, as a result, a hyperfunctional speaking style (Rittich 2018). In this way, the vocal musculature can be damaged (Rittich 2018). There are rooms that have unfavourable hall properties for speakers (Rittich 2018). These are, for example, rooms with straight, smooth walls, large windows and non-textile floor coverings. The reverberation of what is said can mix with the next words, which impairs intelligibility. Speakers have to counteract this by increasing the volume of their speech, usually with additional effort (Rittich 2018). But also the opposite, an acoustic environment that is too anechoic, can be disadvantageous for speakers, because every word in the room is immediately “swallowed” (Rittich 2018). This makes permanent adjustments for the voice and articulation more difficult, which can lead to non-physiological vocal pressure. Similarly, excessively dry air conditions, such as those we experience in many heated seminar rooms, can negatively affect our pharyngolaryngeal mucous membranes, including vocal fold epithelia. They become dry—an extremely unfavourable situation for intensive use of the voice. Drinking water frequently can be a simple remedy to this. Furthermore, a correlation was found between vocal problems and one’s own vocal abilities (Richter and Sandel 2013). If these are low, more vocal problems result. Any healthy voice is capable of being subjected, at times, to unusually intense work for more than 6 to 8 hours a day, or for several days at a time. If performance decreases or even hoarseness sets in, then this is usually a warning sign but can usually be remedied with a few hours or even a few days of vocal rest (Richter and Sandel 2013). If vocal stress becomes permanent for a longer period of time, say, almost every day at work, the voice, which is still in need of rest, is challenged again and again. This leads to the failure of compensation mechanisms, the overload becomes chronic, and functional dysphonia develops (Seidner 2013). This effect is exacerbated if the speech performance is permanently monotonous. Another significant relationship was found between voice problems and high speech rate, high pitch and sustained loudness (Gomes et al. 2018). During intense vocal use, laryngeal and pharyngeal muscles are placed under strong, unaccustomed tension. This, combined with high mental presence and possibly stage fright, results in increased whole-body tone. In the speech process, in which already renewed phonation takes place during the exhalation phase, a breath break is hardly possible. This would be important, however, so that the respiratory muscles are released and the diaphragm in particular has a chance to regenerate briefly. If the rhythmic breathing process is not given, however, this can lead to a muscular imbalance of the respiratory musculature, which in turn triggers unfavourable compensation mechanisms in the vocal musculature. It is supposedly assumed that various job demands (e.g. inattentive students) require a higher or even lower position than one’s primary voice pitch because it is believed a pitch change will gain respect (Tormin and Bock 2018). However, if one does not speak in one’s natural physiological voice pitch in the long run, one risks unfavourable tension conditions in the laryngeal musculature. Prolonged loudness was also a significant influencing variable in the context of a large student population (Gomes et al. 2018). If there is a need for a dynamic increase in intensity to call voice in untrained personnel, especially those with constitutionally unfavourable preconditions, it may lead to compensatory overtonation with a gradual increase of the mean pitch of the speaking voice and a corresponding subjective symptomatology of overload (Rittich 2018).

### Evaluating the influencing variables

When evaluating the influencing variables, a differentiation must be made between, on the one hand, the factors that affect action—such as vocalisation—and, on the other hand, the factors that would call for an adjustment of environmental factors or conditions. For example, the risk factor duration of vocal stress could be addressed both behaviourally by incorporating more individual work into the seminar concept of lecturers, and relatively by offering further training in didactics. Further behavioural preventive measures would be to help those affected to find their own main range of speech and to adapt it adequately to professional requirements (Rittich 2018), and also to avoid a non-physiological posture by first informing lecturers about the connections between posture and voice and then finding ways to improve posture in the relevant speaking situations.

### Consequences of voice problems and starting points for prevention offers

Studies show that university teachers are at risk of developing work-related dysphonia over the course of their professional lives as a result of increased vocal demands (Vilkman 2004; Kooijman et al. 2006). This not only has unfavourable health consequences (voice problems, poorer well-being, more stress) for those affected (Smith et al. 1996) but also negative effects on the learning success of students (due to a loss of quality in teaching) (Rogerson and Dodd 2005) and, in the long run, also on the economy (more sick days, costs for treatment and early retirement) (Verdolini and Ramig 2001). In the worst case, chronic occupational dysphonia can develop, which has not yet been recognised as an occupational disease in Germany (Hammer and Teufel-Dietrich 2017; Kutej 2011). Although the voice is essential for working at a university, surprisingly little is done to preserve it. There is no training or appropriate preparation with regard to the increased vocal stresses in this profession (Boss-Ostendorf and Senft 2018). A congenital voice weakness is unchangeable, but the correct speaking and breathing technique can be learned (Tormin and Bock 2018). This is where an existing discrepancy between professional requirements and insufficient preparation for university teachers in the relevant training and study programmes comes into play (Tormin and Bock 2018). Furthermore, no vocal fitness examination takes place before entering the profession. Hammer and Teufel-Dietrich (2017) emphasise that a vocal fitness examination can reveal a need for prevention. One often searches in vain for offers in the field of prevention and early detection. Appropriate structural and personnel framework conditions should therefore be created in the university setting.

### The International Classification of Functioning, Disability and Health (ICF)

**Fig. 1:**
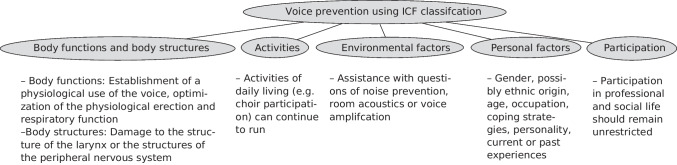
International Classification of Functioning, Disability and Health (ICF) in the context of voice

Prevention would be all the more successful the more it is oriented towards the importance of the professional and personal context. Since the ICF model focuses on strengthening individual resources in everyday life, it therefore applies to the conceptual work of the voice prevention programmes in question (Rittich 2018) (see Fig. 1).

By means of its interrelated components of structure/function, activity, participation and contextual factors, the model takes into account the multidimensional causal structure of occupational dysphonia. In particular, it makes sense to consider the participation component, since the distinguishing feature of the group of professional speakers from other potentially voice-affected people is part of everyday professional life, that is, participation (Ehlert 2011). The holistic perspective on the effects and conditions of voice complaints in university teachers seems particularly suitable for the implementation of preventive intervention measures.

### Aim, research questions and hypotheses

The aim of the study was to identify the group of scientific staff at German universities as suitable recipients for health-promoting offers in the area of voice. It sought to show why this target group, in particular compared to the non-scientific administrative staff also working at universities, would be a preferred candidate for such offers. For this purpose, the effects of voice problems on physical, psychological and functional aspects of the life of university teachers were considered. Subsequently, influencing factors that promote or prevent the development of voice disorders in university teachers were identified.

Finally, possible implications for operational practice at universities have been derived from these findings and measures for prevention and early detection formulated in order to avoid voice disorders in university teachers. In order to examine whether university teachers are more frequently affected by voice problems than university administration employees, whether job-related and individual factors influence the occurrence of voice problems among university teachers and whether there is a connection between voice problems and physical (e.g. more sick days), psychological (more stress, worse well-being/quality of life) or functional (reduction of didactic activities) changes, the following hypotheses were motivated:

1. University teachers report voice problems more often than university administration employees.
2. The more unfavourable job-related conditions prevail, the more frequently university teachers report voice problems.
3. The more unfavourable individual factors prevail, the more frequently university teachers report voice problems.
4. The more symptoms of dysphonia developed, the greater the loss of vocal production.

## Methods

### Study population

The study population included all university faculty and administrators.

### The sample

The sample was a total of 101 people who took part in the questionnaire study in the period from 14 June until 14 July of 2021. Of these, eight participants had to be excluded because they did not meet the inclusion requirement of “belonging to the teaching or administrative staff of a university” in Germany.

### Study participants

**Fig. 2:**
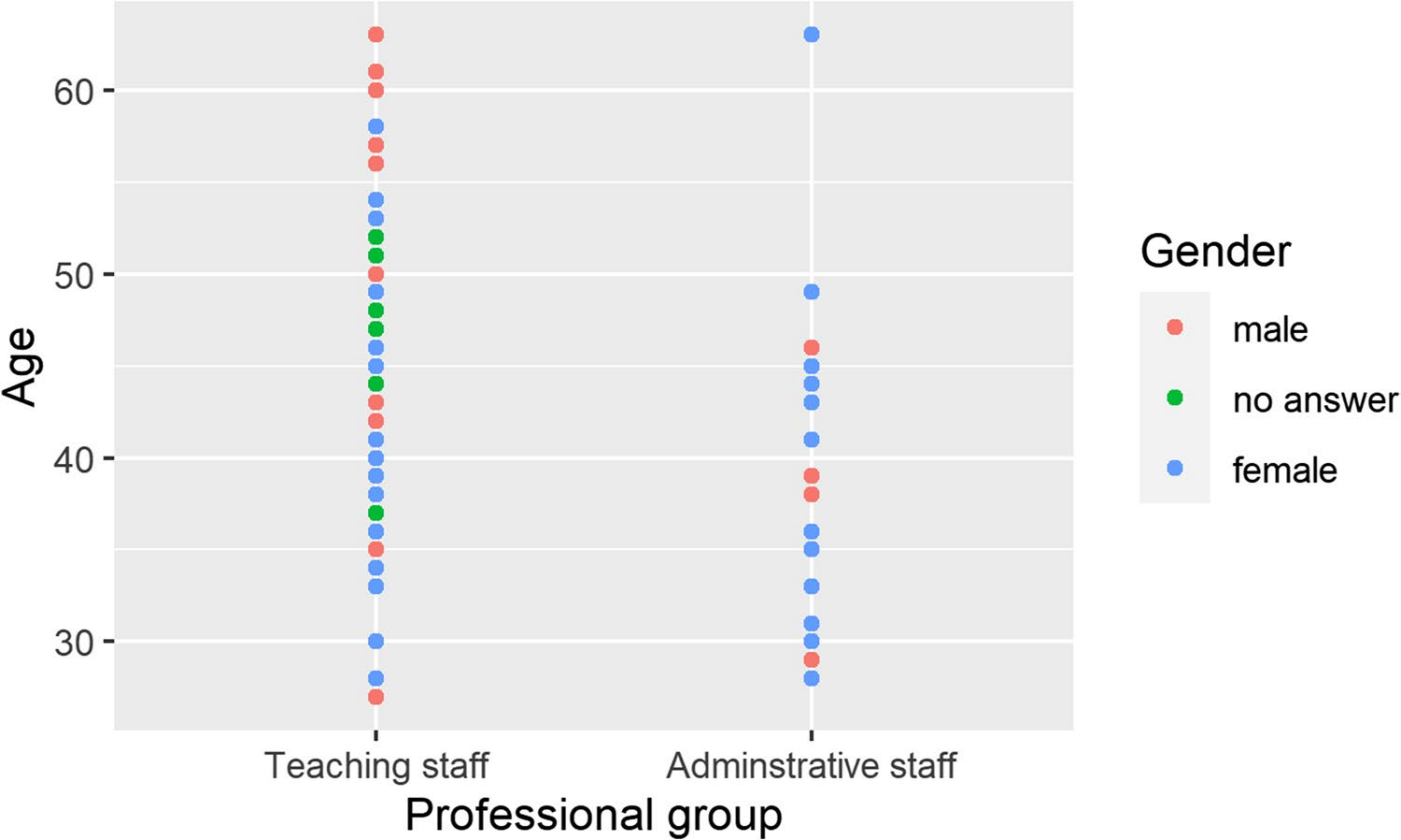
Presentation of the variables occupational group, gender and age

The test data set included the data of 93 participants, which were evaluated with the statistical programme R (https://www.r-project.org/) with the data science package collection tidyverse (Wickham et al. 2018). Fifty-nine participants assigned themselves to female gender and 22 to male gender. Twelve participants did not indicate their gender. The average age of the participants was 49.5 years, ranging from 27 to 65 years. The average age of the male participants was 47 years, and 44.1 years for the female participants; see Fig. 2.

#### Teaching staff

Of the participants, 73 were university teachers. Thirty-six participants (49.3%) from the group of teaching staff stated their position as professors, 30 (41%) as research assistants and seven (9.6%) as teachers for special tasks.

#### Administrative staff

Twenty employees from the human resources administration took part. Six participants (30%) had administrative function, and four (20%) were study programme coordinators. There was one each (5%) of head of student service, site manager, study advisor, study coach, health manager, student services and information technology (IT). Two participants (10%) gave no information about their professional function.

### Study design and procedure

In order to answer the questions and to examine the hypotheses, an empirical-quantitative approach, in the form of a cross-sectional study, was chosen using a questionnaire design. For this purpose, a test group (teaching staff) and a control group (administrative staff) were recruited. The allocation to the groups was made by an initial corresponding question in the questionnaire; see Appendix A. All participants then received the same questions, with the group of teaching staff including additional questions about the teaching situation, previous investigations, training courses and measures or about wishes for the employer. The groups were compared for the presence of voice problems. The groups were then examined to determine whether there is a connection between workplace-related conditions and/or individual factors and the presence of voice problems. The groups were analysed according to whether there is a connection between the severity of symptoms of voice problems and loss of vocal production and the number of sick days.

### Research tool

In order to reach as many potential respondents as possible, a questionnaire in online format was chosen. The online questionnaire was implemented using SoSci Survey (Leiner 2019) and made available to the participants on www.soscisurvey.de after technical and content-related pre-testing.

The theoretical foundation of the questionnaire was based on the application of the International Classification of Functioning, Disability and Health (ICF) model (see introduction).

The questionnaire began with the welcome page; see Appendix A. The participants were informed of the use and the storage of their data. By clicking the continue button, the participants declared their consent. Page 2 asked for socio-demographic information (gender, age, professional group, function and professional experience at the university). The third and fourth pages contained the questions that were aimed exclusively at the teaching staff. These were job-related questions (page 3) such as the size of the student groups, the weekly teaching load, the type and duration of the teaching, the use of technical aids and questions about previous training and studies, whether a voice aptitude test took place before the start of the teaching activity (page 4), whether a voice seminar was attended as part of the university work and whether first-aid measures for the voice are known. The last question asked about wishes to the employer for preserving a good voice. Pages five to ten were aimed at all participants and asked about the voice profile, voice activities with singing or modulation and activity and participation using five-stage multi-category answer formats in the area of voice function. For this, items from existing self-assessment questionnaires were used and supplemented by the specialist literature. This applied to items 14 to 35 (excluding item 17) from the Vocal Profile for Professional Speakers (SPBS) (Ehlert 2011), the studies by Besser et al. (2020), Assuncau et al. (2012), Depolli et al. (2019) and de Medeiros et al. (2012). Item 17 was taken from the Voice Symptom Scale (Deary et al. 2003). The questions on environmental factors on pages 10–11 in multi-category response format (items 36-40) were taken from studies on external risk factors for dysphonia (Vilkman 2004; Kooijman et al. 2006). The items relating to personal factors (items 41 to 48, pages 12 and 13) were taken from the studies by de Almeida et al. (2010) and Alva et al. (2017). Except for question 46, which was asked in mixed response format, all items were created in multi-category response format.

### Data collection

To recruit participants, the author of the study wrote an e-mail to all members of universities in Germany who were in her professional and private network and who worked in the areas of teaching and administration. In addition, the freely accessible e-mail addresses of employees of German universities who met the inclusion criterion of belonging to university staff in the areas of administration or teaching were selected from the corresponding university websites. In addition to the link that led to the questionnaire, the invitation e-mail contained a brief description of the author and the university for which the survey is being carried out. In addition, the questionnaire was briefly presented, and it was explained how the data obtained will be used further. Colleagues proved to be helpful supporters by distributing the invitation to the questionnaire and study via internal university mailing lists and promoting participation in the study (snowball principle).

There was no reward for the participants. The data was entered by the participants according to the input fields in the data mask of the online questionnaire. Data protection was applied in that the data has only been stored in an anonymous form and then processed further. If the purpose of storage is finally no longer applicable, all data will be deleted. The questionnaire could be terminated at any time without giving a reason. By clicking the continue button on the first page of the questionnaire, the participants confirmed their consent to the storage and use of their data as detailed previously on the questionnaire.

## Results

### Working conditions, training and wishes of the teaching staff

Table 1 shows the results of the survey of the teaching staff on their conditions at work, previous examinations/training courses in the field of voice, and their wishes.

**Table 1:** Questions exclusively addressed to teaching staff (*n* = 73) concerning teaching conditions, training and wishes

Item	Variable	Teaching staff (*n* = 73)
Work experience	Average age Span N.A.	10.7 years 1–37 years 2 (2.7%)
Size of the student group	Very small Small Middle Large Very large Others N.A.	31 (42.5%) 47 (64.4%) 19 (26.0%) 3 (4.1%) 3 (4.1%) 4 (5.5%) 25 (34.2%)
Teaching per week on average	Once Twice Three times Four times Daily N.A.	4 (5.5%) 16 (21.9%) 28 (38.4%) 16 (21.9%) 4 (5.5%) 25 (34.2%)
Use of technical aids	Never Rarely Sometimes Often Always N.A.	30 (41.1%) 14 (19.8%) 8 (11.0%) 5 (6.8%) 11 (15.1%) 25 (34.2%)
Type of teaching	Presence Virtual Hybrid All three formats Presence and virtual Presence and hybrid Presence and virtual	44 (60.3%) 52 (71.2%) 30 (41.1%) 18 (24.7%) 21 (28.8%) 1 (1.4%) 13 (17.8%)
Duration of teaching units (TU) at 45 min	2 TU More than 2 TU More than 4 TU More than 6 TU More than 8 TU N.A.	9 (12.3%) 19 (26.0%) 29 (39.7%) 10 (13.7%) 8 (11.0%) 25 (34.2%)
Vocal fitness examination	Yes No N.A.	4 (5.5%) 64 (87.7%) 25 (24.2%)
Training voice	Yes No N.A.	9 (12.3%) 58 (79.5%) 26 (35.6%)
First aid measures for voice	Yes No N.A.	20 (27.4%) 48 (65.8%) 25 (34.2%)
Wishes for the employer	Free input	Offers on voice and relaxation training, practice groups, advanced training, individual coaching, possibility of voice training for doctoral students, medical advice from company doctor, technical support such as headset if masks are required, technical equipment in all teaching rooms, good room insulation, avoid noise from outside/construction noise, longer breaks, optimise distribution of teaching: no 10 units at a time, sensible teaching schedule that avoids more than 4 units per day if possible.

The university teachers surveyed teach predominantly (64.4%) small student groups (21 to 40 people) or very small (42.5%) student groups (1 to 20 people). The majority of the university teachers taught three times a week (38.6%) with generally (39.7%) more than four teaching units per day. At the time of the survey (under pandemic conditions caused by the infectious disease COVID-19), they mostly taught virtually (71.2%) and/or by being present (60.3%), with mostly no (41.1%) to rare (19.2%) use of technical aids such as microphones, audio systems or headsets. Only four (5.5%) of the participants stated that they had undergone a vocal aptitude test with an ENT (ear, nose and throat) doctor/otorhinolaryngologist)/phoniatrist before they began teaching, and 87.7% denied this to be the case. A clear majority (79.5%) stated that they had not completed any further education or training in the field of voice. In addition, 48 (65.8%) of the university teachers surveyed did not know any first-aid measures for the voice. It is therefore not surprising that many concrete wishes were formulated for the university as an employer, which primarily expressed training offers in the areas of voice, relaxation and coaching. In addition, there were requests for medical advice from the company doctor, for optimisation of the technical and acoustic equipment or technical support in all teaching rooms, and for a better distribution of teaching with longer breaks and the avoidance of daily teaching units that exceed four units.

### Vocal profile of teaching and administrative staff

All participants were asked to rate their voice themselves. Table 2 shows the results of the self-declared voice profile.

**Table 2:** Results of the self-assessed voice profile among teaching and administrative staff

Item	Variable	Teaching staff (*n* = 73)	Administrative staff (*n* = 20)	Row sums (*n* = 93)
Voice rough, hoarse, scratchy	Never Rarely Sometimes Often N.A.	8 (11.0%) 33 (45.2%) 23 (31.5%) 3 (4.1%) 6 (8.2%)	7 (35.0%) 11 (55.0%) 2 (10.0%) 0 (0.0%) 0 (0.0%)	15 (16.1%) 44 (47.3%) 25 (26.9%) 3 (3.2%) 6 (6.5%)
Voice away	Never Rarely Sometimes Always N.A.	33 (45.2%) 23 (31.5%) 10 (13.7%) 1 (1.1%) 6 (8.2%)	13 (65.0%) 7 (35.0%) 0 (0.0%) 0 (0.0%) 0 (0.0%)	46 (49.5%) 30 (32.3%) 10 (10.8%) 1 (1.1%) 6 (6.5%)
Uncontrollable voice sound	Never Rarely Sometimes Often Always N.A.	36 (49.3%) 19 (26.0%) 7 (9.6%) 3 (4.1%) 2 (2.7%) 6 (8.2%)	11 (55.0%) 8 (40.0%) 1 (5.0%) 0 (0.0%) 0 (0.0%) 0 (0.0%)	47 (50.5%) 27 (29.0%) 8 (8.6%) 3 (3.2%) 2 (2.2%) 6 (6.5%)
Feeling of mucus in the throat	Never Rarely Sometimes Often Always N.A.	26 (35.6%) 22 (30.1%) 11 (15.1%) 8 (11.0%) 0 (0.0%) 6 (8.2%)	10 (50.0%) 7 (35.0%) 2 (10.0%) 0 (0.0%) 1 (5.0%) 0 (0.0%)	36 (38.7%) 29 (31.2%) 13 (14.0%) 8 (8.6%) 1 (1.1%) 6 (6.5%)
Clearing throat/cough	Never Rarely Sometimes Often Always N.A.	8 (11.0%) 29 (39.7%) 23 (31.5%) 5 (6.8%) 1 (1.1%) 7 (9.6%)	4 (20.0%) 10 (50.0%) 5 (25.0%) 1 (5.0%) 0 (0.0%) 0 (0.0%)	12 (12.9%) 39 (41.9%) 28 (30.1%) 6 (6.5%) 1 (1.1%) 7 (7.5%)
Effort throughout the body	Never Rarely Sometimes Often N.A.	24 (32.9%) 23 (31.5%) 16 (21.9%) 4 (5.5%) 6 (8.2%)	12 (60.0%) 3 (15.0%) 4 (20.0%) 1 (5.0%) 0 (0.0%)	36 (38.7%) 26 (28%) 20 (21.5%) 5 (5.4%) 6 (6.5%)
Voice tone change in the course of the day	Never Rarely Sometimes Often N.A.	12 (16.4%) 29 (39.7%) 19 (26.0%) 7 (9.6%) 6 (8.2%)	7 (35.0%) 7 (35.0%) 4 (20.0%) 2 (10.0%) 0 (0.0%)	19 (20.4%) 36 (38.7%) 23 (24.7%) 9 (9.7%) 6 (6.5%)

Overall, only minor vocal anomalies could be identified. However, the teaching staff, in contrast to the administrative staff, more frequently had problems with the parameters *clearing their throat* and *coughing*. A total of 31.5% of the teaching staff reported difficulties sometimes and 39.7% rarely. For administrative staff, 25% reported problems sometimes and 50% rarely. The parameter *voice rough, hoarse, raspy* was mentioned more frequently (31.5% sometimes and 45.2% rarely amongst teaching staff; and 10% sometimes and 55% rarely for administrative staff). The teaching staff also more frequently (26% sometimes and 39.7% rarely) stated a *change in the sound of the voice over the course of the day* than the administrative staff (35% never and 35% rarely). The parameters from the voice profile *clearing of the throat/cough* and *change in voice tone over the course of the day* were related to age. There was a connection of the effect that clearing the throat, coughing and a change in the tone of the voice over the course of the day among university teaching staff with the increasing of age.

**Fig. 3:**
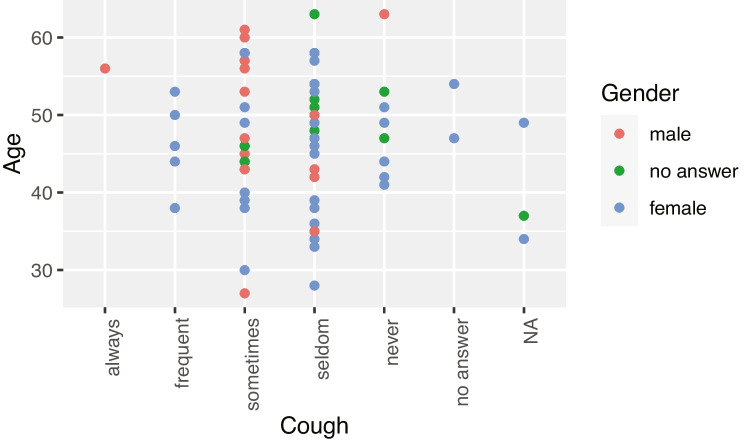
Relationship between the parameters ageing and throat-clearing/coughing among teaching staff

**Fig. 4:**
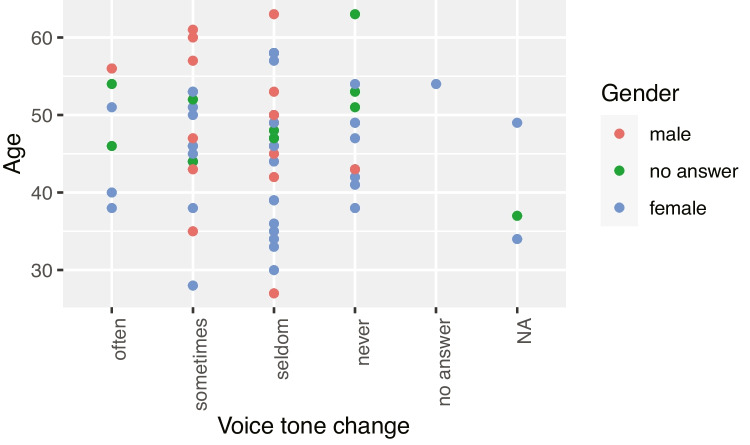
Relationship between the parameters ageing and change in voice pitch over the course of the day among teaching staff

Figures 3 and 4 illustrate that there is a connection to the effect that clearing the throat, coughing and a change in the tone of the voice over the course of the day among university teaching staff increases with age.

#### Vocal activities involving singing and modulation for teaching and administrative staff

Furthermore, the participants were asked about their vocal activities that go hand in hand with singing or vocal modulation. Table 3 illustrates the results.

**Table 3:** Results of the self-assessed voice activities involving singing and modulation among teaching and administrative staff

Item	Variable	Teaching staff (*n* = 73)	Administrative staff (*n* = 20)	Row sums (*n* = 93)
Poor intelligibility on the phone	Never Rarely Sometimes N.A.	42 (57.5%) 20 (27.4%) 5 (6.8%) 6 (8.2%)	11 (55.0%) 8 (40%) 1 (5%) 0 (0.0%)	53 (57.0%) 28 (30.1%) 6 (6.6%) 6 (6.5%)
Problems speaking in front of groups	Never Rarely Sometimes Often N.A.	51 (69.9%) 1 (1.4%) 1 (1.4%) 1 (1.4%) 5 (6.8%)	16 (80.0%) 4 (20.0%) 0 (0.0%) 0 (0.0%) 0 (0.0%)	66 (71.0%) 5 (5.4%) 1 (1.1%) 1 (1.1%) 5 (5.4%)
Does singing cause vocal difficulties	Never Rarely Sometimes Often Always N.A.	31 (42.5%) 17 (23.3%) 11 (15.1%) 5 (6.8%) 1 (1.4%) 8 (11.0%)	11 (55.0%) 8 (40.0%) 3 (15.0%) 0 (0.0%) 0 (0.0%) 0 (0.0%)	42 (45.2%) 25 (26.9%) 14 (15.1%) 5 (5.4%) 1 (1.1%) 8 (8.6%)
Difficulty modulating voice	Never Rarely Sometimes Often N.A.	30 (41.1%) 26 (35.1%) 9 (12.3%) 2 (2.7%) 5 (6.8%)	10 (50.0%) 6 (30.0%) 4 (20.0%) 0 (0.0%) 0 (0.0%)	40 (43.0%) 32 (34.4%) 13 (14.0%) 2 (2.2%) 5 (5.4%)
Long bursts of speech	Never Rarely Sometimes Often Always N.A.	11 (15.1%) 16 (21.9%) 26 (35.6%) 13 (17.8%) 1 (1.1%) 6 (8.2%)	2 (10.0%) 8 (40.0%) 6 (30.0%) 3 (15.0%) 0 (0.0%) 3 (15.0%)	13 (14.0%) 24 (25.8%) 32 (34.4%) 16 (17.2%) 1 (1.1%) 9 (9.7%)
Difficulty adjusting vocally to different dialogue partners and/ or situations	Never Rarely Sometimes Often N.A.	31 (42.5%) 25 (34.2%) 7 (9.6%) 4 (5.5%) 6 (8.2%)	11 (55.0%) 6 (30.0%) 3 (15.0%) 0 (0.0%) 0 (0.0%)	42 (45.2%) 31 (33.3%) 10 (10.8%) 4 (4.3%) 6 (6.5%)
Quick change singing/calling/speaking	Never Rarely Sometimes Often Always N.A.	38 (52.1%) 15 (20.5%) 6 (8.2%) 13 (17.8%) 1 (1.1%) 11 (15.1%)	11 (55.0%) 7 (35.0%) 2 (10.0%) 0 (0.0%) 0 (0.0%) 0 (0.0%)	49 (52.7%) 22 (23.7%) 8 (8.6%) 13 (14.0%) 1 (1.1%) 11 (11.8%)
Problems with certain activities because of voice	Never Rarely Sometimes N.A.	40 (54.8%) 19 (26,.0%) 5 (6.8%) 9 (12.3%)	15 (75.0%) 4 (20.0%) 1 (5.0%) 0 (0.0)	45 (48.4%) 23 (24.7%) 6 (6.5%) 9 (9.7%)

Here, activities in the areas of singing and modulation were hardly affected by vocal influences. However, it was noticeable that the relative majority of university lecturers stated that speaking for a long time is sometimes stressful (35.6%).

#### Vocal activity and participation among teaching and administrative staff

In addition to the questions already asked, there were questions about vocal activity and participation. The results are shown in Table 4. The majority of the people surveyed did not show any impairments in the aforementioned areas.

**Table 4:** Results of vocal activity and participation among teaching and administrative staff

Item	Variable	Teaching staff (*n* = 73)	Administrative staff (*n* = 20)	Row sums (*n* = 93)
Avoid phone	Never Rarely Sometimes N.A.	59 (80.8%) 6 (8.2%) 2 (2.7%) 6 (8.2%)	19 (95.0%) 0 (0.0%) 0 (0.0%) 1 (5.0%)	78 (83.9%) 6 (6.5%) 2 (2.2%) 7 (7.5%)
Avoid groups	Never Rarely N.A.	62 (84.9%) 5 (6.8%) 6 (8.2%)	19 (95.0%) 0 (0.0%) 1 (5.0%)	81 (87.1%) 5 (5.4%) 6 (6.5%)
Avoid singing	Never Rarely Sometimes Often Always N.A.	46 (63.0%) 7 (9.6%) 4 (5.5%) 7 (9.6%) 1 (1.4%) 8 (11.0%)	11 (55.0%) 3 (15.0%) 1 (5.0%) 3 (15.0%) 1 (5.0%) 1 (5.0%)	57 (61.3%) 10 (10.8%) 5 (5.4%) 10 (10.8%) 2 (2.2%) 9 (9.7%)
Avoid expression	Never Rarely Sometimes N.A.	53 (72.6%) 9 (12.3%) 4 (5.5%) 7 (9.6%)	14 (70.0%) 5 (25.0%) 0 (0.0%) 1 (5.0%)	67 (72.0%) 14 (15.1%) 4 (4.3%) 8 (8.6%)
Avoid long speaking engagements	Never Rarely Sometimes Often N.A.	49 (67.1%) 12 (16.4%) 5 (6.8%) 1 (1.1%) 6 (8.2%)	16 (80.0%) 2 (10.0%) 1 (5.0%) 0 (0.0%) 1 (5.0%)	65 (69.9%) 14 (15.1%) 6 (6.5%) 1 (1.1%) 7 (7.5%)
Avoid change of communication partners or situations	Never Rarely Sometimes N.A.	59 (80.8%) 6 (8.2%) 1 (1.1%) 7 (9.6%)	11 (55.0%) 6 (30.0%) 3 (15.0%) 0 (0.0%)	70 (75.3%) 12 (12.9%) 4 (4.3%) 7 (7.5%)
Avoid activities	Never Rarely N.A.	58 (79.5%) 8 (11.0%) 7 (9.6%)	19 (95.0%) 0 (0.0%) 1 (5.0%)	78 (83.8%) 8 (8.6%) 8 (8.6%)

### Influencing environmental factors in teaching and administrative staff

The questionnaire also recorded information on potentially influencing environmental factors at the university; see Table 5. The university teachers reported *background noise* more frequently than the participating administrative staff (32.9% sometimes and 34.2% rarely vs 10% sometimes and 45% rarely), likewise a *dry indoor climate* (37% sometimes and 28.8% rarely vs 20% sometimes and 50% rarely), similarly *poor spatial acoustics* (31.5% sometimes vs 15% sometimes), as well as *missing breaks* (32.9% sometimes vs 10% sometimes).

**Table 5:** Results of environmental factors among teaching and administrative staff

Item	Variable	Teaching staff (*n* = 73)	Administrative staff (*n* = 20)	Row sums (*n* = 93)
Background noise	Never Rarely Sometimes Often Always N.A.	13 (17.8%) 25 (34.2%) 24 (32.9%) 3 (4.1%) 1 (1.4%) 6 (8.2%)	6 (30.0%) 9 (45.0%) 2 (10.0%) 2 (10.0%) 0 (0.0%) 1 (5.0%)	19 (20.4%) 34 (36.6%) 26 (28.0%) 5 (5.4%) 1 (1.1%) 7 (7.5%)
Dry indoor climate	Never Rarely Sometimes Often Always N.A.	9 (12.3%) 21 (28.8%) 27 (37.0%) 7 (9.6%) 1 (1.4%) 6 (8.2%)	3 (15.0%) 10 (50.0%) 4 (20.0%) 3 (15.0%) 1 (5.0%) 1 (5.0%)	12 (12.9%) 31 (33.3%) 31 (33.3%) 10 (10.8%) 2 (2.2%) 7 (7.5%)
Poor spatial acoustics	Never Rarely Sometimes Often Always N.A.	17 (23.3%) 17 (23.3%) 23 (31.5%) 6 (8.2%) 3 (4.1%) 7 (9.6%)	6 (30.0%) 8 (40.0%) 3 (15.0%) 2 (10.0%) 0 (0.0%) 1 (5.0%)	23 (24.7%) 25 (26.9%) 26 (28.0%) 8 (8.6%) 3 (3.2%) 8 (8.6%)
Missing breaks	Never Rarely Sometimes Often Always N.A.	14 (19.2%) 17 (23.3%) 24 (32.9%) 8 (11.0%) 2 (2.7%) 7 (9.6%)	6 (30.0%) 9 (45.0%) 2 (10.0%) 2 (10.0%) 0 (0.0%) 1 (5.0%)	20 (21.5%) 26 (28.0%) 26 (28.0%) 10 (10.8%) 2 (2.2%) 8 (8.6%)
Unphysiological posture	Never Rarely Sometimes Often Always N.A.	29 (39.7%) 19 (26.0%) 10 (14.0%) 4 (5.5%) 2 (2.7%) 9 (12.3%)	8 (40.0%) 6 (30.0%) 4 (20.0%) 1 (5.0%) 0 (0.0%) 1 (5.0%)	37 (39.8%) 25 (26.9%) 14 (15.1%) 5 (5.4%) 2 (2.2%) 10 (10.8%)

### Influencing personal factors in teaching and administrative staff

Finally, individual data of the participants were collected that may have an impact on the voice; see Table 6.

**Table 6:** Results of person-related factors among teaching and administrative staff

Item	Variable	Teaching staff (*n* = 73)	Administrative staff (*n* = 20)	Row sums (*n* = 93)
Nervous and tense	Never Rarely Sometimes N.A.	54 (74.0%) 10 (14.0%) 2 (2.7%) 7 (9.6%)	15 (75.0%) 2 (10.0%) 2 (10.0%) 1 (5.0%)	69 (74.2%) 12 (12.9%) 4 (4.3%) 8 (8.6%)
Shame	Never Rarely Sometimes Often Always N.A.	54 (74.0%) 11 (15.1%) 1 (1.4%) 0 (0.0) 0 (0.0%) 6 (8.2%)	3 (15.0%) 10 (50.0%) 4 (20.0%) 3 (15.0%) 1 (5.0%) 3 (15.0%)	57 (61.3%) 21 (22.6%) 5 (5.4%) 3 (3.2%) 1 (1.1%) 9 (9.7%)
Less competence	Never Rarely Sometimes Often N.A.	56 (76.7%) 7 (9.6%) 2 (2.7%) 1 (1.4%) 7 (9.6%)	17 (85.0%) 1 (5.0%) 1 (5.0%) 0 (0.0%) 1 (5.0%)	73 (78.5%) 8 (8.6%) 3 (3.2%) 1 (1.1%) 8 (8.6%)
Use of medical-therapeutic care	Never Rarely Sometimes N.A.	54 (74.0%) 7 (9.6%) 4 (5.5%) 8 (11.0%)	19 (95.0%) 0 (0.0%) 0 (0.0%) 1 (5.0%)	73 (78.5%) 7 (7.5%) 4 (4.3%) 9 (9.7%)
Sick note	Never Rarely Sometimes N.A.	53 (72.6%) 13 (17.8%) 1 (1.4%) 6 (8.2%)	15 (75.0%) 3 (15.0%) 0 (0.0%) 2 (10.0%)	68 (73.1%) 16 (17.2%) 1 (1.1%) 8 (8.6%)
Smoking	Yes No N.A.	2 (2.7%) 64 (87.7%) 7 (9.6%)	4 (20.0%) 15 (75.0%) 1 (5.0%)	6 (6.5%) 79 (84.9%) 8 (8.6%)
Alcohol intake	Never Rarely Sometimes Often N. A.	6 (8.2%) 41 (56.2%) 19 (26.0%) 1 (1.4%) 6 (8.2%)	1 (5.0%) 10 (50.0%) 8 (40.0%) 0 (0.0%) 1 (5.0%)	7 (7.5%) 51 (54.8%) 27 (29.0%) 1 (1.1%) 7 (7.5%)
Sufficient sleep	Rarely Sometimes Often Always N. A.	8 (11.0%) 12 (16.4%) 38 (52.1%) 9 (12.3%) 6 (8.2%)	2 (10.0%) 6 (30.0%) 9 (45.0%) 2 (10.0%) 1 (5.0%)	10 (10.8%) 18 (19.4%) 47 (50.5%) 11 (11.8%) 7 (7.5%)

Here, 20 (27.4%) university teachers stated that they only sometimes or rarely got enough sleep compared to eight (40%) employees in administration. The remaining parameters were mostly classified as not conspicuous.

### Data processing and statistical analysis

To check the hypotheses, ordinal regression analyses were carried out using the proportional odds (PO) model with the R data science package collection VGAM (version 1.1-5) (Yee 2015).

#### First hypothesis

In order to test the first hypothesis—“University teachers report voice problems more frequently than employees in university administration”—a PO was calculated for the groups—university teachers, administrative employees and the group with missing information—with regard to their voice problems. For this purpose, the items *clearing the throat/cough, voice, rough, hoarse, scratchy and change in voice tone over the course of the day* were used. The results are shown in Table 7.

**Table 7:** Results of the voice profile of teaching and administrative staff determined with ordinal regression analysis using the proportional odds (PO) model

Item	Variable	PO-value	Std. deviation	*t*-value	*p*-value
Clearing throat/cough	Never Rarely Sometimes Always N.A.	−1.15 −0.86 −1.19 −0.34 −17.22	1.55 1.41 1.45 0.00 0.00	−0.74 −0.61 −0.82 −800,760.14 −23,138,556.92	0.46 0.54 0.41 0.00 0.00
Voice rough, hoarse, scratchy	Never Rarely Sometimes N.A.	18.71 17.79 16.08 16.88	0.77 0.64 0.65 0.00	24.38 27.99 24.92 40,143,431.85	0.00 0.00 0.00 0.00
Voice tone change in the course of the day	Never Rarely Sometimes	−0.80 −1.18 −0.76	1.17 1.10 1.13	−0.68 −1.08 −0.68	0.49 0.28 0.50
Teaching staff	N.A.	16.96	1.27	13.34	0.00
Administrative staff	N.A.	16.96	1.27	13.34	0.00
Neither to the teaching nor to the administrative staff	–	38.79	10,065.72	0.00	1.00

The analysis showed a highly significant result for the item *clearing/coughing* (always: *t*-value: −800,760, *p*-value: = 0.00). Compared to administrative staff, university lecturers were more likely to cough and clear their throat when speaking in everyday working life. For the item *voice rough, hoarse, scratchy*: sometimes highly significant results were obtained (for sometimes: *t*-value: 24.92, *p*-value: 0.00; for rarely: *t*-value: 27.99, *p*-value: 0.00; for never: *t*-value: 24.38, *p*-value: 0.00). Compared to administrative staff, university teachers were more often affected by a rough, hoarse or scratchy voice in their everyday work.

#### Second hypothesis

To test the second hypothesis, “The more unfavourable job-related conditions prevail, the more frequently university teachers report voice problems.”, a PO was created for the groups—university teachers, administrative staff and the group with missing information—and carried out with regard to their job-related conditions. For this purpose, the items *dry room climate*, *background noise, poor spatial acoustics* and *lack of breaks* and the items *voice rough, hoarse, scratchy*, *clearing the throat/coughing* and *change in voice tone over the course of the day* were used. The analysis showed highly significant results for the items relevant to the workplace; see Table 8.

**Table 8:** Results of the influences of job-related conditions of teaching and administrative staff determined with ordinal regression analysis using the proportional odds (PO) model

Item	Variable	PO-value	Std. deviation	*t*-value	*p*-value
Dry indoor climate	Never Rarely Sometimes Often N.A.	−52.47 −51.22 −51.70 −52.66 −36.80	0.99 0.75 0.79 1.72 0.00	−53.19 −68.32 −65.21 −30.53 −180,319,075	0.00 0.00 0.00 0.00 0.00
Clearing throat/cough	Never Rarely Sometimes Always N.A.	−1.87 −1.11 −0.90 −0.93 −16.32	2.23 2.17 2.30 0.00 0.00	−0.84 −0.51 −0.39 −10,636,125 −68,017,059	0.40 0.61 0.69 0.00 0.00
Voice rough, hoarse, scratchy	Never Rarely Sometimes N.A.	3.97 2.97 16.08 89.42	0.83 0.64 0.65 0.00	4.76 4.68 24.92 543,544,856.37	0.00 0.00 0.00 0.00
Voice tone change in the course of the day	Never Rarely Sometimes	31.73 31.78 32.75	0.75 0.62 0.79	42.14 51.19 41.6	0.00 0.00 0.00
Background noise	Never Rarely Sometimes Often	71.67 71.04 69.80 72.29	0.87 0.72 0.91 1.59	82.03 99.25 76.88 45.42	0.00 0.00 0.00 0.00
Poor room acoustics	Never Rarely Sometimes Often	14.86 15.08 14.46 15.55	0.92 0.80 0.88 1.65	16.17 18.92 16.37 9.40	0.00 0.00 0.00 0.00
Missing breaks	Never Rarely Sometimes Often	−13.43 −12.91 −15.04 3.60	0.74 0.73 0.99 0.73	−18.21 −17.59 −15.26 4.92	0.00 0.00 0.00 0.00
Teaching staff	N.A.	55.52	0.79	70.50	0.00
Administrative staff	N.A.	55.52	0.79	70.50	0.00
Neither to the teaching nor to the administrative staff	–	13,100,126.36	0.79	16,636,189.82	0.00

In detail, highly significant results were shown for the items *dry indoor climate* (sometimes: *t*-value: −65.21,* p*-value: 0.00; never: *t*-value: −53.19, * p*-value: 0.00, often: *t*-value: −30.53, * p*-value: 0.00, rarely: *t*-value: −68.32, * p*-value: 0.00), *background noise* (sometimes: *t*-value: 76.88, * p*-value: 0.00, never: *t*-value: 82.03, * p*-value: 0.00, often: *t*-value: 45.42, * p*-value: 0.00, rarely: *t*-value: 99.25, * p*-value: 0.00), *poor spatial acoustics* (sometimes: *t*-value: 16.37, * p*-value: 0.00; never: *t*-value: 16.17, * p*-value: 0.00; often: *t*-value: 9.40, * p*-value: 0.00; rarely: *t*-value: 18.92, * p*-value: 0.00) and *missing pauses* (sometimes: *t*-value: −15.26, * p*-value: 0.00; never: *t*-value: −18.21, * p*-value: 0.00; often: *t*-value: 4.92, * p*-value: 0.00; rarely: *t*-value: −17.59, * p*-value: 0.00).

In their day-to-day work, university teachers’ voices were more often than to be expected by chance affected by workplace-related limitations such as poor spatial acoustics, background noise or the lack of breaks.

#### Third hypothesis

In order to test the third hypothesis, “The more unfavourable individual factors predominate, the more frequently university teachers report voice problems”, the item *sufficient sleep* and the items *clearing the throat/cough, rough, hoarse, scratchy voice* and *voice change over the course of the day* were used. The results are shown in Table 9.

**Table 9:** Results of the influences of individual conditions of teaching and administrative staff determined with ordinal regression analysis using the proportional odds (PO) model

Item	Variable	PO-value	Std. deviation	*t* value	*p*-value
Sufficient sleep	Rarely Sometimes Often	0.56 1.63 0.24	1.29 1.11 0.99	0.43 1.48 0.25	0.00 0.00 0.00
Clearing throat/cough	Never Rarely Sometimes Always N.A.	−1.45 −0.88 −1.71 −0.76 −17.12	1.57 1.46 1.57 0.00 0.00	−0.92 −0.60 −1.09 −41,816 −813,733	0.69 0.00 0.00 0.00 0.00
Voice rough, hoarse, scratchy	Never Rarely Sometimes N.A.	18.90 18.14 16.54 17.44	0.93 0.70 0.72 0.00	20.27 26.08 22.98 918,906.04	0.00 0.00 0.40 0.00
Voice tone change in the course of the day	Never Rarely Sometimes	−0.09 −0.66 −0.30	1.50 1.40 1.38	−0.06 −0.47 −0.22	0.00 0.00 0.00
Teaching staff	N.A.	18.16	1.66	10.94	0.00
Administrative staff	N.A.	18.16	1.66	10.94	0.00
Neither to the teaching nor to the administrative staff	–	43.64	1.66	26.29	0.00

The analysis showed a highly significant result for the item *sufficient sleep* (for sometimes: *t*-value: 0.48, *p*-value: 0.00; for often: *t*-value: 0.25, *p*-value: 0.00, for rarely: *t*-value: 0.43, *p*-value: 0.00). Compared to university teachers, administrative employees were less likely to get enough sleep. In order to ascertain whether vocal changes occur as a function of age, the item *age* was also evaluated. With a *t*-value of 2.02 and a *p*-value of 0.04, the values were significant, whereas *gender* was not a significant variable.

In the sample examined, there was a high probability of vocal impairments with increasing age.

#### Fourth hypothesis

In order to test the fourth hypothesis, “The more symptoms of dysphonia appear, the greater the loss of vocal production”, the items *voice rough, hoarse, scratchy, clearing/coughing, change in voice tone over the course of the day* and *long bursts of speech* were used. Table 10 shows the results.

**Table 10:** Results of self-assessed voice profile, voice activities, age and gender among teaching and administrative staff determined with ordinal regression analysis using the proportional odds (PO) model

Item	Variable	PO-value	Std. deviation	*t*-value	*p*-value
Clearing throat/cough	Never Rarely Sometimes Always N.A.	−1.10 −0.72 −1.77 1.60 −17.88	1.77 1.58 1.71 0.00 0.00	−0.62 −0.46 −1.04 11,336,196.4 −77,392,521	0.53 0.65 0.30 0.00 0.00
Voice rough, hoarse, scratchy	Never Rarely Sometimes N.A.	21.02 20.50 18.55 17.70	0.92 0.83 0.96 0.00	22.84 24.71 19.28 149,963,324.36	0.00 0.00 0.00 0.00
Voice tone change in the course of the day	Never Rarely Sometimes	−0.55 −1.42 −1.04	1.55 1.48 1.46	−0.35 −0.96 −0.71	0.72 0.34 0.47
Long bursts of speech	Never Rarely Often	−2.91 −1.75 −1.74	0.99 0.81 0.87	−2.93 −2.16 −1.99	0.00 0.03 0.05
Age	-	−0.09	0.04	−2.02	0.04
Gender	Female N.A.	0.04 −17.24	0.80 0.00	0.05 −120,697,624	0.96 0.00
Teaching staff	N.A.	13.35	1.87	7.13	0.00
Administrative staff	N.A.	13.35	1.87	7.13	0.00
Neither to the teaching nor to the administrative staff	–	37.94	1.87	20.25	0.00

For the item *cough/clear your throat* there was a highly significant result (always: *t*-value: 11,336,196.39, *p*-value: 0.00). Compared to administrative staff, university teachers were more likely to have vocal impairments in their everyday work, such as clearing their throats or coughing. In addition, highly significant results were shown for the parameters *Rough, hoarse, raspy voice* (sometimes: *t*-value: 19.28, *p*-value: 0.00; rarely: *t*-value: 24.71, *p*-value: 0.00) and *long bursts of speech* (sometimes: *t*-score: −3.55, *p*-value: 0.00; never: *t*-score: −2.93, *p*-value: 0.00; rarely: *t*-score: −2.16, *p*-value: 0.00).

## Discussion

### University teachers report voice problems more often than employees in university administration

The first hypothesis was confirmed by the results of the items *clearing/coughing* and *voice rough, hoarse, raspy*. According to this, in the sample examined, university teachers are affected by voice problems more often than administrative employees in their professional environment. Based on proven differences in the two aforementioned items, the hypothesis of the equality of conditions can be refuted. The results thus confirmed the literature (e.g. Higgins 2006) which found an increased prevalence of voice disorders in university teachers due to excessive speaking load. However, the absence of a significant difference in the item *change in voice over the course of the day* must not be interpreted as evidence that the two groups have no differences. The non-existence of differences on this item could not be proven beyond doubt.

### The more unfavourable workplace-relevant conditions prevail, the more frequently university teachers report voice problems

The second hypothesis could be confirmed by the parameters *dry room climate, poor room acoustics, background noise* and *lack of breaks* based on the available data with the available test power. Unfavourable conditions in the workplace, such as a dry room climate, poor spatial acoustics, background noise and the lack of breaks, are more prevalent in the group of university teachers that are vocally impaired compared to administrative employees. The results pointed in the same direction as the findings by Korn et al. (2015) who reported an association between hoarseness among teaching staff and their work environment in terms of volume, sound level and poor air quality. The results relating to the connection between voice problems and poor spatial acoustics were also confirmed in the literature by Rittich (2018). Previously, the findings by Gomes et al. (2018) have shown that voice overload can have adverse consequences. The results of the examined sample pointed in a similar direction, since university teachers more than randomly mentioned missing breaks as the cause of their voice problems.

### The more unfavourable individual factors prevail, the more frequently university teachers report voice problems

The third hypothesis could be verified in the examined sample with the existing test power for the parameter age. *Age* is a sensitive factor that favoured the development of a voice disorder. Age-related, physiological changes in the voice apparatus can no longer be adequately compensated for by additional factors. This is confirmed by previous work on age-related changes and their consequences for members of speaking professions (Tormin and Bock, 2018). One of the additional factors could be the stress factor, which was not explicitly asked about in this survey. Findings by Korn et al. (2015) have confirmed a connection between stress, nervousness and vocal problems in university teachers.

### The more symptoms of dysphonia present, the greater the loss of vocal production

The fourth hypothesis agreed with the study data for the items *cough/clearing throat, hoarse, hoarse, raspy voice* and *long vocal onsets*. This was also confirmed by the results of Rittich (2018), who found a compensatory over-toning with a gradual increase in the middle speaking voice range and associated subjective symptoms of overload in the direction of vocal fatigue, especially in untrained personnel. The majority of the sample examined was voice untrained.

### Study limitations and strengths

This study had several limitations that constrain its generalisation. First of all, it was based only on an online questionnaire. Due to the pandemic conditions which prevailed at the time the study was undertaken, no clinical evaluation was performed with the subjects who participated in the study.

Second was the small random sample size of the group of administrative staff, which comprised only 20 participants. According to Ioannidis and Lau (1997), studies with a small number of subjects only provide unambiguous answers to research questions if the study is well defined and the population is homogeneous. A possible solution would be a collection of various small groups that are as homogeneous as possible. That offers the opportunity to test different groups and differently defined studies in order to create tailor-made offers, such as in training/consulting. For this, the recruitment channels and the wording of the invitation text would have to have been reconsidered. Presumably, members of the administrative staff per se participate less in scientific studies and therefore need different information or incentives to motivate them to participate.

Third, the group of university staff stated that they mostly taught with small or very small groups of students. This can lead to a selection bias due to a lower speaking load, as could be the case in larger groups of students. As a result, this could systematically have distorted the results with regard to the information on the vocal profile, vocal activity, especially with singing, modulation and participation. University teachers with larger groups of students should therefore be enabled to be included in a targeted manner in departments with more heavily frequented courses.

In addition, there was another form of distortion: the loss bias due to omissions of answers. This happened up to 26 times in several questions. This could easily be counteracted by designing the online questionnaire in such a way that participants are reminded of unanswered questions, or filling it out is mandatory in order to be able to continue processing the questionnaire.

There may also have been a measurement bias due to systematic differences in the way the result parameters were collected. So, for example, only two questions were asked related to stress (whole-body exertion and jittery, tight voice). Detailed questions about the current stress level, general well-being, psychological stress, self-regulation strategies and self-efficacy would have been beneficial here, as there is an evident connection between psychological condition, emotional state and voice. Numerous studies show a relationship between functional dysphonia and psychological characteristics or disorders such as anxiety, stress and depression. For example, almost 30% of patients with functional dysphonia showed psychiatric abnormalities (Stapleton et al. 2020; White et al. 2012; Dietrich et al. 2008). In addition, a connection has been shown between certain previous illnesses, their medication (reflux disease, depression, hearing loss, respiratory diseases, severe general diseases, hormonal disorders) and the occurrence of voice disorders. To this end, questions about previous illnesses and any medication intake could have been included in the list of questions for this study.

Despite the aforementioned limitations, the study had several strengths. In fact, the questionnaire mainly included short questions, and the scales were frequently used in the reviewed literature and were validated outside Germany. The study included 93 university professionals, which increased the precision of the outcomes and might have ensured a good representativeness of the German university teaching professionals, especially professors and lecturers, which may have improved the generalisability of the results. However, due to the limited time frame of the project, the ideal sample size could not be achieved. The ideal sample size was calculated using Qualtrics' sample calculator (https://www.qualtrics.com/de/erlebnismanagement/marktforschung/stichprobenrechner/) and given with a confidence level of 95%, a population size of 400.100 and a margin error of 5%, an ideal sample size of 384 probands. Therefore, the results cannot be transferred to the population of the sample.

Moreover the results should be interpreted cautiously, because all data referred to Germany. Generalisation to other countries with comparable academic conditions is conceivable and would have to be examined accordingly.

Finally, additional research should be conducted using other methods, for example, longitudinal studies or qualitative studies, in order to support the findings.

## Conclusions

The study confirmed that some job-related and some individual factors influence the occurrence of voice disorders in university teachers in Germany (and qua generalisability in other countries with comparable academic conditions) and, therefore, requires further research. Unfavourable conditions at the workplace (dry room climate, background noise, poor spatial acoustics and lack of breaks) and increasing age increased the voice problems of university teachers in the sample tested. The fact that university teachers with voice problems reduced their use of voice compared to university teachers without voice problems, and thus their didactic activities and were therefore more often ill and physically and mentally impaired, could not be confirmed on the basis of the data collected. Nevertheless, such a correlation could theoretically exist.

The results showed a clear need, because on the one hand, only a very small number (5.5%) of the university teachers surveyed took part in a voice aptitude test before starting their job, and most of them (79.5%) had not attended any further education or training in the field of voice at the time of the survey. In addition, 65.8%, that is, more than half of the participants, stated that they had no knowledge of first-aid measures for the voice. On the other hand, requests were made for training and coaching offers, for advice from the company doctor, for optimisation of the technical and acoustic equipment or for technical support in all teaching rooms to be addressed. In addition, there were requests for optimisation with regard to a better distribution of teaching with longer breaks and daily teaching that do not exceed four units in a row.

Of further research interest would be to devote attention to already established voice training programmes. It is still largely an open research question as to which programmes have a lasting effect on the clientele of university teachers. In addition, workplace health promotion and occupational safety in the German university are more closely interlinked, the quality of prevention offers is ensured through binding standards, and the role of doctors (Bocken et al. 2015) and therapists in prevention is strengthened. Existing health and early detection examinations (not just an annual eye test, but also a voice strain screening) should also be established and individualised stress and risk factors increasingly taken into account. For insured persons with special occupational stresses, such as increased speech stress, it should be easier to take advantage of preventive services.

## Supplementary information

ESM 1 Questionnaire (translated in English, conducted in German) (PDF 208 kb)

## Data Availability

Not applicable
