# Supplementary material for: Prevention of occupational dysphonia in scientific university personnel: A cross-sectional study
Source: Z Gesundh Wiss. 2023 Jan 13:1–18. Online ahead of print. doi: 10.1007/s10389-022-01805-0 (PMC9836919; doi:10.1007/s10389-022-01805-0)
Supplement: Supplementary file 1 — Questionnaire (translated in English, conducted in German) (PDF 208 kb) [file 10389_2022_1805_MOESM1_ESM.pdf]

WELCOME!

Please take a few minutes.Thank you for your willingness to participate in my questionnaire study on the topic: **„Prevention of voice disorders in university personel".**

The data you enter will be stored according to the input fields. The responsible person is Mrs. Christiane Lücking. Your data will only be passed on to third parties in anonymous form for scientific purposes. Should the purpose of the storage has finally ceased to exist, all your data will be deleted. For the rest the Federal Data Protection Act applies. Cancellation of the questionnaire is possible at any time without giving reasons.

By clicking on the button below (Continue) you declare your consent to the storage and use of the data in this questionnaire.

SOCIODEMOGRAPHICS AND OCCUPATION

1. **First of all, we would like to ask you for some information about yourself.**

You are: ◻ Male
◻ Female
◻ Diverse

How old are you? ____ years

2. **To which professional group of the university do you belong?**

◻ Teaching university staff

◻ Administrative university staff

◻ Neither teaching nor administrative university staff

3. **Please specify your function:**

◻ Professor

◻ Research assistant

◻ Others: _________

4. **Please indicate your work experience at the university.**

_____ years

QUESTIONS ONLY FOR TEACHING STAFF

5. **How large are the groups of students you teach?**
   □ very small (1 bis 20 persons)
   □ small (21 bis 40 persons)
   □ middle (41 bis 60 persons)
   □ large (61 bis 80 persons)
   □ very large (81 bis 100 persons)
   □ others (which?): ___________

6. **How many times a week do you usually teach (on average)?**
   □ one time a week
   □ two times a week
   □ three times a week
   □ four times a week
   □ daily a week
   □ others: ________

7. **Do you use technical aids (microphone, audio system, headset) for your teaching?**
   □ never
   □ rarely
   □ sometimes
   □ often
   □ always

8. **How do you teach?**

   □ being present

   □ virtual

   □ hybrid solution (virtual and present)

9. **How long do you usually teach in a day? (Duration of teaching units (TU) á 45 min)**

□ 2 TU

□ more than 2 TU

□ more than 4 TU

□ more than 6 TU

□ more than 8 TU

□ others: ______

10. Have you had a vocal aptitude test with an ENT doctor (Ear Nose and Throat Doctor)/ Otorhinolaryngologist/ Phoniatrist before you began teaching?

□ Yes
□ No

11. Have you attended in further education or training in the field of voice within your university work?

□ Yes. Which? _______________
□ No

12. Do you know so-called "first aid measures" for the voice?
□ Yes. Which? ________________
□ No. I am not aware of any.

13. What would you like your employer to do to preserve your good voice?

Please enter your ideas and suggestions here. Use one per line per comment for this.
____
____

VOICE PROFILE

14. Does your voice sound unpleasantly rough, hoarse or scratchy?
Please refer to your daily work routine only!

□ never
□ rarely
□ sometimes
□ often
□ always

15. Does your voice suddenly fail you when you speak?
Please refer to your daily work routine only!

□ never
□ rarely
□ sometimes
□ often
□ always

16. Do you feel like the sound of your voice is uncontrollable?
Please refer to your daily work routine only!

□ never
□ rarely
□ sometimes
□ often
□ always

17. Do you have the feeling of mucus in your throat?
Please refer to your daily work routine only!

□ never
□ rarely
□ sometimes
□ often
□ always

18. Do you have to clear your throat or cough when you speak?
Please refer to your daily work routine only!

□ never
□ rarely
□ sometimes
□ often
□ always

19. Do you feel general strain throughout your body when you speak?
Please refer to your daily work routine only!

- □ never
- □ rarely
- □ sometimes
- □ often
- □ always

20. Does the sound of your voice change over the course of the day? Please refer to your daily work routine only!

- □ never
- □ rarely
- □ sometimes
- □ often
- □ always

VOICE ACTIVITIES WITH SINGING AND MODULATION

21. Are you difficult to understand on the phone?
Please refer to your daily work routine only!

□ never
□ rarely
□ sometimes
□ often
□ always

22. Do you have problems speaking in front of groups because of your voice?
Please refer to your daily work routine only!

□ never
□ rarely
□ sometimes
□ often
□ always

23. Does singing cause you vocal difficulties?
Please refer to your daily work routine only!

□ never
□ rarely
□ sometimes
□ often
□ always

24. Do you have difficulty modulating the sound of your voice?
Please refer to your daily work routine only!

□ never
□ rarely
□ sometimes
□ often
□ always

25. Do you feel that speaking for a long time is a strain on your voice?
Please refer to your daily work routine only!

□ never
□ rarely
□ sometimes
□ often
□ always

26. Do you find it difficult to adapt vocally to different conversational partners and / or situations?
Please refer to your daily work routine only!

□ never
□ rarely
□ sometimes
□ often
□ always

27. Does the rapid change from singing or calling and speaking cause you difficulties?
Please refer to your daily work routine only!

□ never
□ rarely
□ sometimes
□ often
□ always

28. Do you have problems performing certain activities because of your voice?
Please refer to your daily work routine only!

□ never
□ rarely
□ sometimes
□ often
□ always

ACTIVITY AND PARTICIPATION

29. Do you avoid making phone calls because of your voice?
Please refer to your daily work routine only!

□ never
□ rarely
□ sometimes
□ often
□ always

30. Do you avoid speaking in front of groups because of your voice?
Please refer to your daily work routine only!

□ never
□ rarely
□ sometimes
□ often
□ always

31. Do you avoid singing because of your voice?
Please refer to your daily work routine only!

□ never
□ rarely
□ sometimes
□ often
□ always

32. Do you avoid situations that require special vocal expression because of your voice?
Please refer to your daily work routine only!

□ never
□ rarely
□ sometimes
□ often
□ always

33. Do you avoid long speaking engagements because of your voice?
Please refer to your daily work routine only!

□ never
□ rarely
□ sometimes
□ often
□ always

34. Do you avoid changing communication partners and/or situations because of your voice?
Please refer to your daily work routine only!

□ never
□ rarely
□ sometimes
□ often
□ always

35. Do you avoid certain activities that your job actually requires because of your voice?
Please refer to your daily work routine only!

□ never
□ rarely
□ sometimes
□ often
□ always

ENVIROMENTAL FACTORS

36. Is background noise at work affecting your voice?
Please refer to your daily work routine only!

□ never
□ rarely
□ sometimes
□ often
□ always

37. Does speaking in rooms with dry air at work affect your voice?
Please refer to your daily work routine only!

□ never
□ rarely
□ sometimes
□ often
□ always

38. Do poor spatial acoustics in the workplace affect your voice?
Please refer to your daily work routine only!

□ never
□ rarely
□ sometimes
□ often
□ always

39. Is a lack of vocal rest breaks at work affecting your voice?
Please refer to your daily work routine only!

□ never
□ rarely
□ sometimes
□ often
□ always

40. Does your posture at work affect your voice?
Please refer to your daily work routine only!

□ never
□ rarely
□ sometimes
□ often
□ always

PERSONAL FACTORS

41. Are you nervous and tense about your voice when you speak at work?
Please refer to your daily work routine only!

□ never
□ rarely
□ sometimes
□ often
□ always

42. Are you ashamed of your voice?
Please refer to your daily work routine only!

□ never
□ rarely
□ sometimes
□ often
□ always

43. Do you feel less competent at work because of your voice?

□ never
□ rarely
□ sometimes
□ often
□ always

44. Have you already sought medical-therapeutic help for voice problems?

□ never
□ rarely
□ sometimes
□ often
□ always

45. Have you had to call in sick due to voice problems?

□ never
□ rarely
□ sometimes
□ often
□ always

46. Do you smoke?
□ Yes, how many cigarettes usually a day?

____

□ No, I do not smoke.

47. Do you drink alcohol?

□ never
□ rarely
□ sometimes
□ often
□ always

48. Can you sleep well and enough?

□ never
□ rarely
□ sometimes
□ often
□ always

END

Thank you very much for your cooperation! I will be happy to inform you about the results of the study on request. Simply send me an e-mail.

Kind regards, Christiane Lücking
